# Noninvasive Management after a Traumatic Anterior Knee Dislocation in a Cruciate Retaining Total Knee Arthroplasty: A Case Report

**DOI:** 10.1055/s-0043-1770978

**Published:** 2024-04-22

**Authors:** Rodrigo Sattamini Pires e Albuquerque, Thiago Vivacqua, José Leonardo Rocha de Faria, Douglas Mello Pavão, Victor Elias Titonelli

**Affiliations:** 1 Instituto Nacional de Traumatologia e Ortopedia, Rio de Janeiro, RJ, Brasil; 2 Clínica de Medicina Esportiva Fowler Kennedy, Ontário, Canadá

**Keywords:** arthroplasty, replacement, knee, knee dislocation, postoperative complications

## Abstract

The reported case describes a traumatic anterior knee dislocation of a previous asymptomatic knee after a posterior cruciate-retaining primary knee arthroplasty. The described patient accidentally rolled over her knee six years after the surgical intervention. Anterior traumatic dislocation after knee arthroplasty is an uncommon event often leading to prosthetic's components revision due to its associated ligament injuries. A noninvasive approach was successfully achieved with temporary external fixation and a short period using a rigid knee brace.

## Introduction

Total knee arthroplasty (TKA) is a highly successful intervention achieving high patient satisfaction rates, long-term implant survivorship, and low an incidence of reoperation. 1 2 However, early or late postoperative complications can lead to unsatisfactory clinical outcomes. 1 Late 70's, Insall and coworkers were the first author to report atraumatic posterior knee dislocation (KD) in a series of 220 TKAs. 3

Knee dislocation after TKA often occurs in a posterior direction in a posterior stabilized (PS) prosthesis due to a cam-post mechanism failure or associated with posterior cruciate ligament (PCL) insufficiency in a cruciate retaining (CR) TKA. 1 In contrast, anterior dislocation of the knee was rarely reported and associated with vascular injury. 2 4 5 6 7 8 Moreover, KD after TKA frequently leads to a ligament injury and joint instability. 1 2 4 8 Therefore, a complete revision arthroplasty is generally necessary to achieve a stable joint.

This manuscript describes a case of anterior KD six years after a primary CR-TKA. Our goal was to report radiologic and clinical results with noninvasive management. The proposed method achieved satisfactory clinical outcome without a revision arthroplasty.

## Case Presentation

**Fig. 1:**
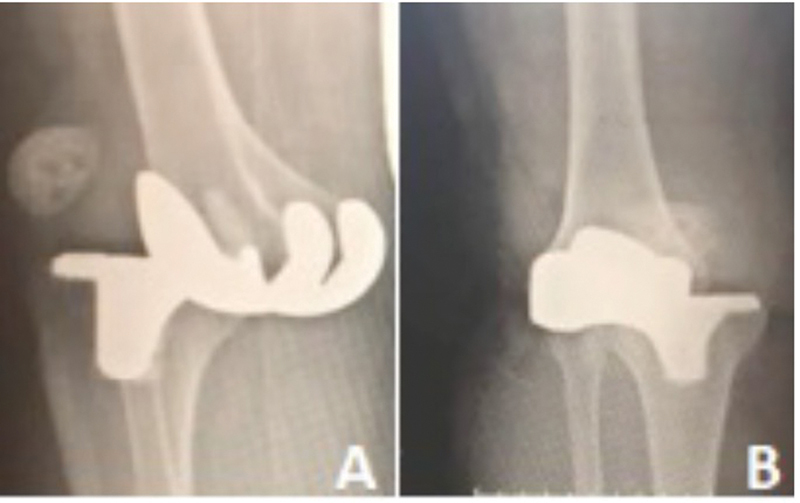
Anteroposterior pre reduction radiographic assessment ( **A** ). Lateral pre reduction radiographic assessment ( **B** ).

A 60's years woman came to the emergency department reporting an episode of collapse and fall leading to a traumatic anterior KD. A cruciate-retaining primary knee replacement was performed in her right knee 6 years forward. Her previous medical record was unremarkable. Additionally, she was performing daily activities without any limitations prior to the traumatic event. Immediately after the reported trauma, severe knee pain and significant joint effusion were noticed. By then, the patient was immediately removed to an operating room in a regional hospital. At the radiologic assessment a complete anteromedial KD was confirmed ( Fig. 1A-B ).

At the clinical examination, complete peroneal nerve palsy and loss of sensibility distal to the knee were reported. No vascular changes were noticed given a symmetrical and palpable dorsalis pedis and posterior tibial pulses. Moreover, a doppler ultrasonography was performed showing an intact arterial blood flow distally to the popliteal artery.

**Fig. 2:**
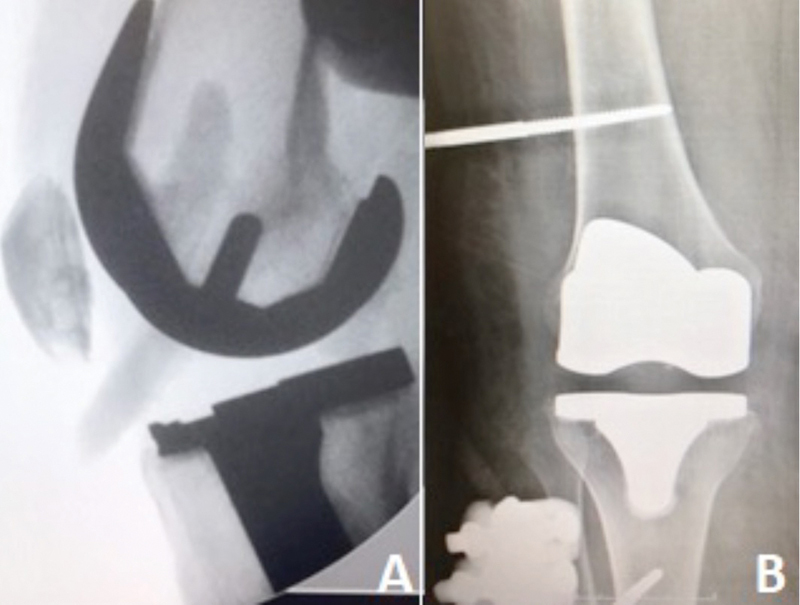
Lateral post reduction radiographic assessment ( **A** ). Anteroposterior post reduction radiographic assessment ( **B** ).

The orthopedic surgeon performed an under-spinal anesthesia manipulation and closed prosthesis reduction 4 hours after the admission time. Intraoperative fluoroscopy showed a well-reduced prosthetic joint without any associated fracture or images suggesting prosthetic component failure ( Fig. 2A-B ). After the reduction, a second doppler ultrasonography was performed. No vascular deficit was identified, and a symmetrical lower limb pulse was confirmed. Any additional clinical examination or stress radiologic test was performed.

## Treatment

**Fig. 3:**
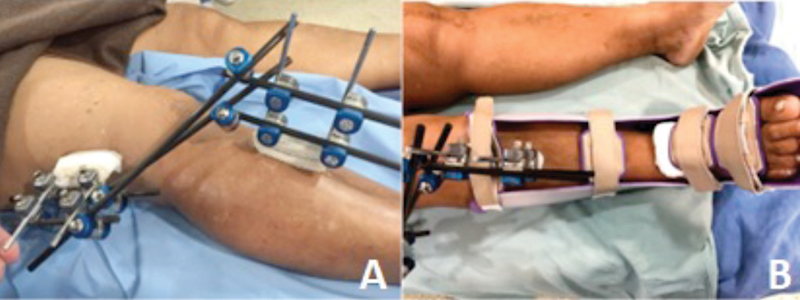
Postoperative trans articular external fixation ( **A** ). Ankle-foot orthoses applied before patient discharge ( **B** ).

The limb was settled in extension with an external fixator after joint reduction ( Fig. 3A ). An ankle-foot orthosis was placed to avoid a foot droop ( Fig. 3B ). The external fixator was removed 3 weeks postoperatively. After that, a rigid knee brace was placed, and as tolerated weight bearing was suggested with the brace locked in extension. A normal sensitive and motor fibular nerve function was diagnosed at three months post-reduction. At the same moment, full weight-bearing was allowed, and the brace support was discontinued.

**Fig. 4:**
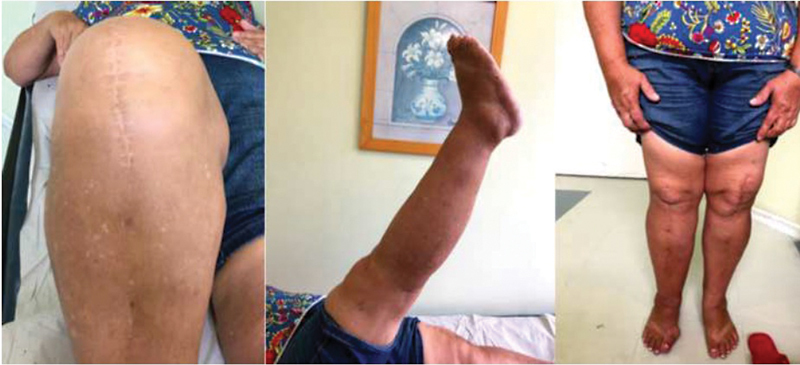
Two years follow-up postoperative clinical evaluation.

**Fig. 5:**
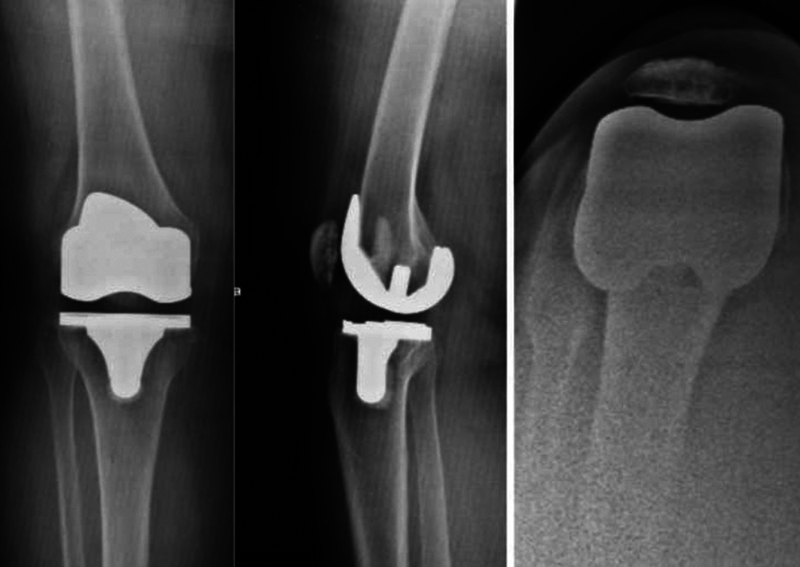
Two years follow-up post reduction radiographs.

Two years after the traumatic KD no residual joint swelling was identified, and a range of motion of about 0-110° was achieved. No extension or flexion instability was identified at clinical examination ( Fig. 4 ). Regardless of the traumatic PCL failure no objective knee instability symptoms were reported. No stress radiologic analysis was performed due to the lack of instability symptoms ( Fig. 5 ).

## Discussion

A case of traumatic anterior KD in an asymptomatic CR TKA was reported. Reviewing its operative notes, no issues were reported during the primary surgical intervention. A functional and stable prosthetic joint was achieved after a trans-articular external fixation followed by a rigid knee brace. Our group believes that the remained PCL fibers and capsular tear healed properly given the acceptable clinical outcome during the reported period. Additionally, the rigid brace leads to achieving stable knee and medial side ligament healing.

Traumatic anterior TKA-KD is uncommon and generally associated to a medial collateral ligament tear or PCL insufficiency in a CR prothesis. Posterior dislocation is often associated to can-post mechanics failure in a posterior stabilized TKA. 1 2 A few cases of anterior KD have been reported often associated to additional knee injuries, vascular changes, and joint instability. 2 4 5 6 7 8 An inappropriate flexion-extension gap balance was suggested as a risk factor for KD after TKA. 8

A high incidence of neurovascular complications can be observed after TKA-KD. 5 6 7 For this reason, early closed reduction is strongly recommended. Our patient had peroneal palsy 3 months after the reduction intervention. Addevico et al. 5 published a similar case involving an atraumatic anterior KD 6 years after TKA. The reported case developed a lower limb arterial thrombosis needing an arterial bypass ending with an unrecovered neurologic deficit. Ahn et al. 4 observed that neurologic changes heal around six months after the injury. Similar to the reported cases, Villanueva and coworkers reported a case of anterior KD with a peroneal palsy, from which the patient recovered completely. 8

Moser and coworkers suggested a diagnostic algorithm in order to recognize PCL insufficiency after CR-TKA. The same author suggested that an onset of new anterior knee pain might indicate an excessive tibial posterior translation in insufficient PCL 9 Additionally, a SPECT/CT was suggested seeking to a patellar bone tracer uptake. A patellar overload can suggest an abnormal posterior tibial translation. 10

Based on our reported case, we recommend immediate close reduction, comprehensive vascular assessment followed for trans-articular external fixator for three weeks followed for rigid brace locked in extension to walk. We understand that every case should be individually addressed based on its primary intraoperative report, radiologic assessment, and clinical examination. A revision knee arthroplasty with more constrained implants should be consider in cases of symptomatic and objective signs of knee instability after TKA-KD.

The reported case showed a satisfactory clinical outcome without a revision arthroplasty after a short period of trans articular external fixation followed by a rigid knee brace support after a traumatic anterior knee dislocation in a CR-TKA.
