# Supplementary material for: Noninvasive Management after a Traumatic Anterior Knee Dislocation in a Cruciate Retaining Total Knee Arthroplasty: A Case Report
Source: Rev Bras Ortop (Sao Paulo). 2024 Apr 22;59(Suppl 2):e155–8. doi: 10.1055/s-0043-1770978 (PMC11679635; doi:10.1055/s-0043-1770978)
Supplement: Related texts

# Research article: Tratamento não invasivo após luxação traumática anterior do joelho em artroplastia total de joelho com preservação do ligamento cruzado posterior: Relato de caso

**Keywords:** artroplastia do joelho, complicações pós-operatórias, luxação do joelho

## Abstract

O caso relatado descreve uma luxação anterior traumática do joelho que era assintomática após uma artroplastia primária da articulação com preservação do ligamento cruzado posterior. A paciente descrita rolou acidentalmente sobre o joelho 6 anos após a intervenção cirúrgica. A luxação anterior traumática após artroplastia do joelho é um evento incomum, muitas vezes levando à revisão dos componentes protéticos devido às lesões ligamentares associadas. Uma abordagem não invasiva, composta por fixação externa temporária e um curto período de uso de joelheira rígida, foi realizada com sucesso.

## Introdução

A artroplastia total do joelho (ATJ) é uma intervenção muitíssimo bem-sucedida, com altas taxas de satisfação do paciente e sobrevida do implante em longo prazo e baixa incidência de reoperação. 1 2 No entanto, complicações pós-operatórias precoces ou tardias podem gerar desfechos clínicos insatisfatórios. 1 No final dos anos 1970, Insall e colaboradores foram os primeiros a relatar a luxação posterior atraumática do joelho em uma série de 220 ATJs. 3

A luxação do joelho após a ATJ geralmente ocorre em direção posterior em uma prótese estabilizada posterior (PE) devido a uma falha do mecanismo *cam-post* ou associada à insuficiência do ligamento cruzado posterior (LCP) em uma ATJ de preservação do ligamento cruzado posterior. 1 Por outro lado, a luxação anterior do joelho foi relatada com maior raridade e associada à lesão vascular. 2 4 5 6 7 8 Além disso, a LP após a ATJ tende a causar lesão ligamentar e instabilidade articular. 1 2 4 8 Assim, uma artroplastia de revisão completa é frequentemente necessária para obtenção de uma articulação estável.

Este manuscrito descreve um caso de luxação anterior do joelho anterior 6 anos após uma ATJ-PLCP. Nosso objetivo foi relatar os desfechos radiológicos e clínicos do tratamento não invasivo. O método proposto teve desfecho clínico satisfatório sem artroplastia de revisão.

## Apresentação do Caso

Uma mulher de 60 anos deu entrada no pronto-socorro relatando um episódio de colapso e queda que causou uma luxação anterior traumática do joelho. Uma artroplastia primária do joelho com preservação do ligamento cruzado posterior havia sido realizada em seu joelho direito 6 anos antes. Seu histórico médico prévio era normal. Além disso, ela realizava atividades diárias sem quaisquer limitações antes do evento traumático. Logo após o trauma relatado, notou-se dor intensa no joelho e derrame articular importante. A essa altura, a paciente foi imediatamente levada para o centro cirúrgico de um hospital regional. A avaliação radiológica confirmou a luxação anteromedial completa do joelho ( Fig. 1A-B ).

**Fig. 1:** Avaliação radiográfica anteroposterior pré-redução ( **A** ). Avaliação radiográfica lateral pré-redução ( **B** ).

Ao exame clínico, havia paralisia completa do nervo fibular e perda da sensibilidade distal ao joelho. Não foram observadas alterações vasculares, com pulsos simétricos e palpáveis da artéria pediosa dorsal e tibial posterior. Além disso, uma ultrassonografia com Doppler demonstrou o fluxo sanguíneo arterial intacto distal à artéria poplítea.

O cirurgião ortopedista realizou manipulação sob raquianestesia e redução fechada da prótese 4 horas após a internação. A fluoroscopia intraoperatória mostrou uma articulação protética bem reduzida sem qualquer fratura associada ou imagens que sugerissem falência da prótese ( Fig. 2A-B ). Depois da redução, uma segunda ultrassonografia com Doppler foi realizada e não identificou nenhum déficit vascular, além de confirmar o pulso simétrico do membro inferior. Outros exames clínicos ou radiológicos de estresse foram realizados.

**Fig. 2:** Avaliação radiográfica lateral pós-redução ( **A** ). Avaliação radiográfica anteroposterior pós-redução ( **B** ).

## Tratamento

O membro foi colocado em extensão com fixador externo após a redução da articulação ( Fig. 3A ). Uma órtese tornozelo-pé foi colocada para evitar o desenvolvimento de síndrome do pé caído ( Fig. 3B ). O fixador externo foi removido 3 semanas após a cirurgia. Em seguida, uma imobilizador longo do joelho rígida foi colocada e, conforme tolerado, foi sugerida a sustentação do peso com a órtese travada em extensão. A função sensorial e motora do nervo fibular era normal 3 meses após a redução. Nesse momento, a carga total foi permitida e o uso da órtese foi interrompido.

**Fig. 3:** Fixação externa transarticular pós-operatória ( **A** ). Órtese tornozelo-pé colocada antes da alta da paciente ( **B** ).

Dois anos após a luxação traumática do joelho, não havia edema articular residual e a amplitude de movimento era de cerca de 0 a 110°. Nenhuma instabilidade de extensão ou flexão foi identificada no exame clínico ( Fig. 4 ). Independentemente da falência traumática do LCP, não houve relato de nenhum sintoma objetivo de instabilidade do joelho. A análise radiológica de estresse não foi realizada devido à ausência de sintomas de instabilidade ( Fig. 5 ).

**Fig. 4:** Avaliação clínica pós-operatória aos 2 anos de acompanhamento.

**Fig. 5:** Radiografias pós-redução aos 2 anos de acompanhamento.

## Discussão

Relatamos um caso de luxação anterior traumática do joelho em ATJ-PLCP assintomática. Não havia relato de nenhum problema no prontuário da intervenção cirúrgica primária. A articulação protética era funcional e estável após a fixação externa transarticular seguida pelo uso de imobilizador longo do joelho rígido. Nosso grupo acredita que as fibras remanescentes do LCP e a ruptura capsular cicatrizaram adequadamente devido ao desfecho clínico aceitável durante o período relatado. Além disso, a órtese rígida leva à cicatrização estável do joelho e do ligamento lateral medial.

A ATJ-luxação anterior traumática do joelho é incomum e, de modo geral, associada a uma ruptura do ligamento colateral medial ou insuficiência do LCP em uma prótese de RC. A luxação posterior é frequentemente associada à falência da mecânica *cam-post* em uma ATJ posterior estabilizada. 1 2 Alguns casos de luxação anterior do joelho, frequentemente associados a outras lesões no joelho, alterações vasculares e instabilidade articular, foram relatados. 2 4 5 6 7 8 O desequilíbrio do espaço em flexão-extensão foi sugerido como fator de risco para a luxação do joelho após a ATJ. 8

Há uma alta incidência de complicações neurovasculares após a ATJ-luxação do joelho. 5 6 7 Por isso, a redução fechada precoce é altamente recomendada. Nossa paciente apresentava paralisia peroneal 3 meses após a intervenção da redução. Addevico et al. 5 publicaram um caso semelhante de luxação anterior atraumática do joelho 6 anos após a ATJ. Este paciente desenvolveu trombose arterial de membro inferior com necessidade de *bypass* arterial e, por fim, apresentou déficit neurológico sem recuperação. Ahn et al. 4 observaram que as alterações neurológicas se resolveram em torno de 6 meses após a lesão. De maneira semelhante, Villanueva e colaboradores relataram um caso de luxação anterior do joelho com paralisia fibular e recuperação completa do paciente. 8

Moser e colaboradores sugeriram um algoritmo diagnóstico para reconhecimento da insuficiência do LCP após a ATJ-RC. Os mesmos autores sugeriram que o surgimento de uma nova dor anterior no joelho pode indicar uma translação posterior excessiva da tíbia na presença de insuficiência do LCP. 9 Além disso, uma tomografia computadorizada (TC) por emissão de fóton único (SPECT)/TC foi sugerida para determinação da incorporação de marcador na patela. A sobrecarga patelar pode indicar a translação posterior anormal da tíbia. 10

Com base em nosso relato, recomendamos redução fechada imediata, avaliação vascular abrangente, uso de fixador externo transarticular por 3 semanas e, a seguir, uso de órtese rígida travada em extensão para deambulação. Entendemos que cada caso deve ser tratado individualmente conforme o avaliação inicial intraoperatória, a avaliação radiológica e o exame clínico. Uma artroplastia de revisão do joelho, com implantes mais restritos, deve ser considerada em casos de sinais sintomáticos e objetivos de instabilidade do joelho após a ATJ-luxação do joelho.

O caso relatado teve desfecho clínico satisfatório sem necessidade de artroplastia de revisão após um curto período de fixação externa transarticular seguida pelo uso de imobilizador longo do joelho rígido rígida após uma luxação anterior traumática do joelho em uma ATJ-RC.
